# Human Urinary Kallidinogenase decreases recurrence risk and promotes good recovery

**DOI:** 10.1002/brb3.1033

**Published:** 2018-07-20

**Authors:** Dong Han, Xin Chen, Dongmei Li, Shuang Liu, Yi Lyu, Juan Feng

**Affiliations:** ^1^ Department of Neurology The Affiliated Shengjing Hospital of China Medical University Shenyang Liaoning China; ^2^ Department of Neurology Shanghai Fifth People's Hospital Fudan University Shanghai China; ^3^ Department of Medical Affairs Techpool Bio‐pharma Co., Ltd Guangzhou Guangdong China

**Keywords:** acute ischemic stroke, Human Urinary Kallidinogenase, recurrence

## Abstract

**Objectives:**

To evaluate the efficacy of Human Urinary Kallidinogenase (HUK) for secondary stroke prevention in the treatment of acute ischemic stroke (AIS) patients.

**Materials and Methods:**

In this retrospective study, from October 2016 to June 2017, 300 consecutive AIS patients were registered in our database. Among them, 145 patients received HUK treatment (HUK group), and 155 patients received basic treatment (control group). Basic treatment was administrated on all patients. 0.15 PNA unit of HUK injection plus 100 ml saline in intravenous infusion was performed in the HUK group, with once a day for 14 consecutive days. The rate of recurrent stroke and modified Rankin Scale (mRS) scores in two groups were analyzed 12 months after the treatment.

**Results:**

No difference was found in the age, gender, comorbidities, smoking history, and NIHSS scores between two groups before treatment (*p* > 0.05). 12 months after treatment, 10 patients in the HUK group (10.3%) and 26 patients in the control group (16.8%) got stroke recurrence at 12 months (*p* = 0.009). The mRS score of HUK group was significantly lower than that in the control group (2.3 ± 1.2 vs. 3.5 ± 1.4, *p* = 0.011).

**Conclusion:**

Human Urinary Kallidinogenase is able to reduce the risk of stroke recurrence and promote good recovery for AIS patients within 12 months.

## 1 INTRODUCTION

Nowadays, the risk of stroke recurrence is still high worldwide. A study in the United States showed that the incidence of stroke recurrence was 185,000 in 795,000 cases (23.3% per year; Go et al., 2013). Another study obtained data from the Swedish Stroke Register between 1998 and 2009 reported that 11.3% of 196 765 patients with ischemic stroke had a recurrent ischemic stroke within 1 year (Bergström et al., 2017). Results from the China National Stroke Registry from 2007 to 2008 showed that the recurrence rate of patients with acute ischemic stroke (AIS) was nearly 20% at 12 months (Wang et al., 2013).

Human Urinary Kallidinogenase (HUK), a glycoprotein extracted from men's urine, is a kallikrein–kinin system regulating medicine. HUK has been listed as a state category I new drug for the treatment of stroke patients by China's State Food and Drug Administration. Many studies showed that HUK has therapeutic effect for AIS (Wu, Lyu, Zhong, Liu, & Liu, 2017). Animal studies have indicated that HUK could inhibit the decrease of cerebral blood flow and suppress brain edema and block poststroke inflammatory cascades (Chen et al., 2010). However, whether HUK is efficient in preventing stroke recurrence of patients with AIS has not been reported yet. Therefore, we conducted a retrospective, registration‐based study to assess the effects of HUK on preventing stroke recurrence within 1 year in AIS patients.

## 2 METHODS

### 2.1 Study population

From October 2016 to June 2017, we retrospectively collected data of 300 consecutive AIS patients who were admitted in the neurology department of our hospital. They were in accordance with the diagnostic criteria of cerebral infarction approved by the fourth national cerebrovascular academic conference (1995). Among them, 145 patients received HUK treatment plus basic treatment were taken as study subjects, Inclusion criteria for cases: (a) age ranging from 18 to 80 years; (b) patients with the first onset; (c) onset time <72 hr; (d) stroke confirmed by head CT or MRI; (e) patients without incomplete hepatic and renal function or severe psychotic disease; (f) patients without history of hemorrhagic stroke, brain tumor, and brain trauma.

### 2.2 Therapeutic methods

Basic treatment was performed among all patients according to disease condition, with therapy of antiplatelet, statins, neuroprotection, dehydrator, controlling blood pressure, and blood glucose. Hundred and forty‐five patients were included in HUK group who received 0.15 PNA unit of HUK (Trade name: Kailikang, Guangdong Techpool Bio‐pharma Co., Ltd. With approved medicine of H20052065) injection plus 100 ml saline in intravenous infusion, with once a day for 14 consecutive days. 155 patients were included in control group who received basic treatment only.

### 2.3 Study design

This was a retrospective, single‐center, and registry‐based study. We obtained the data of patients from our database system and divided them into two groups according to the treatment they received. Information about the baseline characteristics (gender, age, comorbidities included diabetes hypertension and hyperlipoidemia, and history of smoking), the National Institute of Health Stroke Scale (NIHSS) scores before treatment, the 12‐month modified Rankin Scale (mRS) by telephone follow‐up and the rate of recurrence at 12 months were recorded and compared.

### 2.4 Statistical analyses

Statistical analyses were performed using SPSS20.0 software (IBM SPSS, Armonk, NY, USA). The mean ± standard deviation (*SD*) was used to express the continuous variables. The categorical variables were expressed by number and percentage. The student's *t* test or Fisher's exact test was used for continuous variables and the Chi‐squared test was used for categorical variables. *p* < 0.05 was considered to be statistically significant.

## 3 RESULTS

### 3.1 Baseline characteristics

There were 83 males and 62 females in the HUK group, with an average age at 72.3 ± 12.5 years old. Among them, 43 patients had diabetes, 52 patients with hypertension, 79 patients with hyperlipoidemia. There were 47 patients had history of smoking. The NIHSS score before treatment in the HUK group was 5.7 ± 1.8. In the control group there were 86 males and 69 females with an average age at 72.1 ± 11.4 years old. Fifty‐eight patients had diabetes, 64 patients with hypertension, 83 patients with hyperlipoidemia. Forty‐seven patients had history of smoking and the NIHSS score before treatment was 5.5 ± 1.9. No statistically significant difference in baseline characteristics was found between the two groups (*p* > 0.05; Table 1).

**Table 1:** Basic characteristics of all patients

	HUK group (*n* = 145)	Control group (*n* = 155)	*p* value
Age (year, *x* ± s)	72.3 ± 12.5	72.1 ± 11.4	0.908
Male, *n* (%)	83 (57.2)	86 (55.5)	0.759
Diabetes, *n* (%)	43 (29.6)	58 (37.4)	0.155
Hypertension, *n* (%)	52 (35.9)	64 (41.3)	0.335
Hyperlipoidemia, *n* (%)	79 (54.5)	83 (53.5)	0.871
Smoking, *n* (%)	47 (32.4)	51 (32.9)	0.928
NIHSS score before treatment (*x* ± s)	5.3 ± 1.8	5.5 ± 1.9	0.735

*Note*

HUK: Human Urinary Kallidinogenase; NIHSS: National Institute of Health Stroke Scale.

### 3.2 Laboratory determinations of patients in two groups on 14th day

No significant difference in laboratory determinations were found between two groups after 14‐day treatment (P_WBC_ = 0.531, P_CRP_ = 0.614, P_LDL_ = 0.389, P_Triglyceride_ = 0.383, P_Total cholesterol_ = 0.759; Table 2).

**Table 2:** Laboratory determinations of patients in two groups on 14th day

	HUK group (*n* = 145)	Control group (*n* = 155)	*p* value
WBC count (×10^9^/L)	7.90 ± 3.36	8.03 ± 2.80	0.531
CRP (mg/L)	3.90 ± 12.06	4.02 ± 4.35	0.614
LDL (mmol/L)	2.12 ± 1.44	2.75 ± 0.85	0.389
Triglyceride (mmol/L)	4.54 ± 1.74	4.85 ± 1.09	0.383
Total cholesterol (mmol/L)	1.09 ± 0.28	1.19 ± 0.29	0.759

*Note*

CRP: C‐reactive protein; LDL: low‐density lipoprotein cholesterol; WBC: white blood cell.

### 3.3 Efficacy and safety of HUK

Ten patients in the HUK group (10.3%) and 56 patients in the control group (16.8%) got stroke recurrence at 12 months (*p* = 0.009). Twelve‐month mRS scores of the HUK group and the control group were 2.3 ± 1.2 and 3.5 ± 1.4 respectively (*p* = 0.011). No adverse consequence was reported in the HUK group (Table 3).

**Table 3:** Outcomes stroke recurrence of all patients at 12 months

	HUK group (*n* = 145)	Control group (*n* = 155)	*p* value
Stroke recurrence at 12‐month, *n* (%)	10 (10.3)	26 (16.8)	0.009
12‐month mRS score (*x* ± s)	2.3 ± 1.2	3.5 ± 1.4	0.011

*Note*

HUK: Human Urinary Kallidinogenase; mRS: modified Rankin Scale.

## 4 DISCUSSION

This retrospective study showed that patients treated with HUK had a decreased risk for stroke recurrence within 1 year compared with controls. In addition, HUK significantly promoted favorable recovery in AIS patients compared with those without HUK during the 1‐year follow‐up. Similar effects of HUK have been observed in other studies (Ding, Lu, Ding, Su, & Chen, 2007).

The history of ischemic stroke carries a strong risk for ischemic stroke recurrence and some studies showed the mortality of AIS patients with a history of ischemic stroke was higher as compared with those without prior ischemic stroke (Kubo et al., 2006; Lip, Nieuwlaat, Pisters, Lane, & Crijns, 2010). Kallikreins, an important component of kallikrein–kinin system which has been shown to have a protective effect on patients with ischemic stroke (Zhang, Tao, Liu, & Wang, 2012). Kallikrein is a member of the serine proteinase superfamily, and a number of studies have reported its functions which include increasing regional cerebral blood flow by dilating arterioles in the ischemic area selectively, inhibiting cell apoptosis and inflammatory reaction and promoting angiogenesis and neurogenesis (Ling et al., 2008; Lu et al., 2008; Stone et al., 2009; Xia et al., 2006). However, whether HUK, a commercially available kallikrein–kinin system regulating medicine, is efficient in preventing recurrent stroke has not been reported yet. Previous studies have demonstrated that antiplatelet agents, statins, warfarin, and diabetic agents could reduce risk of recurrent stroke for specific stroke patient (Adams et al., 2008; Prasad, Kaplan, & Passman, 2012; Rother & Crijns, 2010; Sacco et al., 2008). In this study, we firstly found that implementation of HUK during initial hospitalization after acute onset was effective for preventing recurrent stroke within 12 months.

It was reported that lowering blood pressure was an effective measure to prevent stroke recurrence. The American Heart Association/American Stroke Association stated that a reduction in stroke recurrence has been associated with an average lowering of 10/5 mmHg (Feldstein, 2014). Hypertension is a strong risk factor for stroke recurrence by damaging endothelial cell. Kallikrein protein infusion had the ability to improve neurological function directly (Chao & Chao, 2006). The kallikrein–kinin system could be activated by HUK (Sahan et al., 2006) and transfer kininogen hydrolysis into kinin and kallidin to release nitric oxide (NO) relaxing vascular smooth muscle (Perilli et al., 2012). Moreover, the expression of vascular endothelial growth factor and its receptor could be reduced by kinins which was another component of kallikrein–kinin system, then it could enhance angiogenesis (Ke & Jing, 2016). These findings may explain the mechanism under the effect of HUK preventing recurrent stroke. We did not find difference in laboratory factors after 14‐day treatment between two groups, this might because the inflammation conditions of patients in two groups were improved after 14‐day treatment and returned to normal level.

In addition, we also found patients with HUK treatment had a lower mRS at 12‐month, which meant HUK could promoted favorable recovery in AIS patients. In animal studies, the inflammatory cell accumulation could be inhibited by kallikrein in the ischemic brain. Furthermore, after the stimulatory effect of kinin on neuronal cell proliferation, it has been confirmed that kallikrein enhanced angiogenesis and promoted neurogenesis after the stimulatory effect of kinin on neuronal cell (Lip et al., 2010). In clinical studies, researchers also found that HUK could improve favorable recovery in AIS patients with level 3 hypertension within 3 months through its property of selectively dilating arterioles in the ischemic area and promoting the formation of new blood vessels (Zhang et al., 2012). Our findings were in accordance with these results. Moreover, we did not find side effect of HUK treatment in this study on patients, which proved that HUK treatment was safe to prevent ischemic stroke recurrence and promote good recovery for AIS patients.

Several limitations should be considered when interpreting the results of this study. First, patients in this study were grouped without randomization, and all patients were from one medical center. Second, the12‐month follow‐up is probably too short to observe in full scale the therapeutic effects of HUK. Third, we did not analyze whether different dose of HUK had different impact on the therapeutic effect and we did not record the changes of factors (such as blood pressure and laboratory determinations) at 12 months. Therefore, a multicenter placebo controlled randomized study is needed due to the limitation of the small sample size of this study.

In conclusion, the HUK treatment for ischemic stroke patients may reduce the risk of stroke recurrence and promote good recovery for AIS patients within 12 months.

## CONFLICT OF INTEREST

The authors report no conflict of interest.
